# Ganglion Impar Block and Pulsed Radiofrequency Under Fluoroscopic Guidance in Chronic Coccydynia: A Retrospective Case Series

**DOI:** 10.7759/cureus.97753

**Published:** 2025-11-25

**Authors:** Joana Saldanha, Mariana Crispiniano, José Luís Carvalho

**Affiliations:** 1 Department of Physical Medicine and Rehabilitation, Unidade Local de Saúde da Região de Aveiro, Aveiro, PRT; 2 Department of Physical Medicine and Rehabilitation, Centro de Reabilitação do Norte, Vila Nova de Gaia, PRT

**Keywords:** chronic pain, coccydynia, coccyx pain, fluoroscopy, ganglion impar, interventional pain procedures, nerve block, pulsed radiofrequency treatment, sacrococcygeal region, treatment outcome

## Abstract

Introduction

Chronic coccydynia is characterized by persistent pain in the coccygeal region and can significantly impair functionality and quality of life. While most patients respond to conservative treatments, some experience refractory pain that may benefit from minimally invasive interventions, such as ganglion impar block and/or pulsed radiofrequency (PRF). This study aims to investigate the effectiveness and safety of fluoroscopy-guided ganglion impar block and/or PRF in patients with chronic coccydynia refractory to conservative treatments.

Methods

We conducted a retrospective case series involving 24 patients with coccydynia lasting at least three months, who underwent ganglion impar block and/or PRF between 2021 and 2025. Clinical data - namely, demographics, comorbidities, history of trauma, vaginal delivery or tumours, sacrococcygeal joint dysfunction, and pain intensity measured with the Numerical Rating Scale (NRS) before and at one, three, and six months post-procedure - were collected and analysed descriptively.

Results

Our case series included 24 patients (91.7% females), who were naïve to minimally invasive treatments for coccydynia, with a mean age of 46.1 ± 15.9 years and a mean BMI of 24.2 ± 5.3 kg/m². Of the total 24 patients, 14 (58.3%) underwent a ganglion impar block. These patients presented a baseline mean NRS of 8.4 ± 1.4 and reported a mean pain reduction of 64% at one month after the procedure, 60% at three months, and 50% at six months. On the other hand, 10 (41.7%) patients were submitted to PRF of the ganglion impar after an immediate positive response to the ganglion impar block. These patients presented a baseline mean NRS of 8.5 ± 1.4 and yielded mean pain reductions of 73% at one month, 62% at three months, and 49% at six months. Three (12.5%) patients with comorbid anxiety and depression experienced meaningful pain relief, regardless of the procedure performed. Patients whose severe coccydynia returned after initial relief underwent repeat ganglion impar block or PRF; repeated procedures showed diminished efficacy, with lower pain relief observed at six months compared to the initial intervention (mean pain reductions of 21% and 38% at six months, respectively). All interventions were performed safely, with no reported complications.

Conclusion

Fluoroscopy-guided ganglion impar block and PRF appear to be safe and effective initial interventions for managing chronic coccydynia refractory to conservative treatment. Our data demonstrated clinically meaningful and similar pain relief for up to six months with both procedures. Modest pain relief was observed with repeated ganglion impar block or PRF. Further research with larger patient cohorts is warranted to determine optimal subsequent options, for example, thermal radiofrequency of the ganglion impar or sacrococcygeal nerves, or chemical neurolysis, following an initial response to a ganglion impar block or PRF.

## Introduction

Coccydynia, or coccygodynia, is a multifactorial condition characterized by pain in the coccygeal region [1]. It predominantly affects females and individuals with obesity [2,3]. It is often caused by direct trauma to the coccyx (falling backwards onto the buttocks), repetitive microtrauma (prolonged sitting on hard or uncomfortable surfaces), and childbirth, particularly difficult or instrument-assisted vaginal deliveries using forceps or vacuum devices [1-4]. Nontraumatic causes may also occur, including sacrococcygeal joint dysfunction (degenerative changes, coccygeal hyper- or hypomobility, or subluxation) [1,4]. Anatomical variations of the coccyx (e.g., markedly curved coccyx) may also contribute [4].

Coccydynia may also be attributed to referred pain from musculoskeletal conditions (such as lumbar disc herniation, sacroiliac joint dysfunction, and piriformis syndrome), pelvic floor dysfunction (namely myofascial pain involving the coccygeus muscle, levator ani, and the anococcygeal ligament), visceral pelvic pathologies (including rectal, sigmoid colon, or urogenital disorders), or neuropathic causes such as pudendal neuralgia [1,3]. Referred pain may occur due to neural convergence at second-order neurons in the dorsal horn of the spinal cord, encompassing viscerosomatic mechanisms [1,3]. Other less common causes include infection, tumours, or inflammatory conditions such as ankylosing spondylitis. Idiopathic coccydynia occurs when no precise precipitating event or pathology is identified [1].

Coccydynia is frequently comorbid with clinically significant anxiety and depression [5]. This co-occurrence may exacerbate pain perception and catastrophizing, contributing to chronicity, increased pain-related disability, and functional impairment [1,5]. It is also associated with a lower response to treatment [6].

Coccydynia is often underdiagnosed, leading to delayed treatment and an increased risk of pain chronification and associated psychosocial morbidity [5,7]. The exact incidence of coccydynia is unknown, which further complicates epidemiological understanding and resource allocation.

Most patients respond to conservative treatments, such as non-steroidal anti-inflammatory drugs (NSAIDs), physiotherapy programs oriented by a physiatrist, paracetamol, tramadol, and psychotherapy for comorbid anxiety and depression [7].

Refractory cases to conservative measures may benefit from minimally invasive interventions since coccygectomy has limited outcomes and substantial risk of severe complications [7,8].

Ganglion impar (Walther’s ganglion) is a solitary sympathetic structure located anterior to the sacrococcygeal joint. It relays sympathetic and visceral afferent input from the distal rectum, perineum, urethra, vulva, and coccyx, making it a strategic target for pain relief interventions [8]. Ganglion impar block has demonstrated efficacy in managing coccygeal pain of both benign and malignant causes [9-12]. Several studies demonstrated significant reductions in pain scores at six months after ganglion impar corticoanesthetic block, particularly in patients with a history of coccygeal trauma [9-12]. Literature suggests that pulsed radiofrequency (PRF) of the ganglion impar, performed after a diagnostic block, can provide clinically meaningful pain relief in patients with chronic coccydynia refractory to conservative management [13]. However, high-quality randomized controlled trials in interventional procedures for coccydynia management are still lacking.

In this case series, we present the demographics and clinical findings of 24 patients submitted to corticoanesthetic block and/or PRF of the ganglion impar for chronic coccydynia. This study aims to investigate the effectiveness and safety of fluoroscopy-guided ganglion impar block and/or PRF for the treatment of chronic refractory coccydynia. Given the nature of the study, the objectives are primarily descriptive, and patients are examined across different subgroups. We aim to assess the magnitude of pain reduction and the duration of symptomatic improvement. To achieve this, subgroup analyses will compare outcomes between patients receiving only a corticoanesthetic block versus those undergoing PRF of the ganglion impar. We also intend to evaluate the benefit of repeated procedures - both repeated blocks and repeated PRF - and to explore the impact of psychiatric comorbidities on treatment response. This case series addresses an existing gap in the literature regarding the therapeutic value of ganglion impar interventions for chronic coccydynia, especially in light of the limited effectiveness of more invasive surgical approaches. 

## Materials and methods

Study design and patient selection

A retrospective data analysis was conducted using the electronic medical records of all fluoroscopy-guided procedures performed at the Intervention and Musculoskeletal Rehabilitation Unit of the Centro de Reabilitação do Norte (CRN), Vila Nova de Gaia, Portugal, between October 2021 and January 2025. We selected all patients presenting with coccydynia lasting more than three months who underwent ganglion impar block and/or PRF at the Intervention and Musculoskeletal Rehabilitation Unit. Informed consent was obtained from all patients. Relevant demographic and clinical data were extracted and reviewed. To ensure data accuracy and completeness, telephone interviews were performed to verify medical records and obtain additional information when necessary, in order to complete all the variables we intended to analyse. Given the small sample size, only descriptive analyses were performed.

Inclusion and exclusion criteria

Patients were included if they presented with coccygeal pain for more than three months and if they were submitted to a ganglion impar block and/or PRF. Patients were excluded if they had incomplete records or had loss to follow-up.

Data collection

Clinical data were collected retrospectively, including sex, age, comorbidities, weight, height, history of tumours, depression or anxiety, tenderness on external palpation in the coccygeal region, prior sacrococcygeal trauma, sacrococcygeal joint dysfunction (degenerative or mechanical changes, such as sacrococcygeal osteoarthritis; coccygeal hypermobility, hypomobility, or subluxation), history of vaginal delivery, including instrument-assisted deliveries (forceps or vacuum), treatments, timing, and adverse events or complications. Pain intensity was measured using the Numerical Rating Scale (NRS, 0-10, where 0 indicates no pain and 10 indicates worst imaginable pain), a validated and widely used tool for pain measurement [14]. NRS was measured before the procedure and at one, three, and six months post-procedure. 

A ganglion impar block was executed as the initial step to identify if meaningful pain relief (defined as more than 50% reduction in NRS pain score) was reported. Subsequently, some patients underwent PRF.

Procedures were conducted on an outpatient basis without sedation and were consistently well tolerated. After obtaining written informed consent, patients were positioned prone. Interventions were carried out under strict sterile technique. All procedures were performed by a single physiatrist, experienced in fluoroscopy-guided techniques.

**Figure 1:**
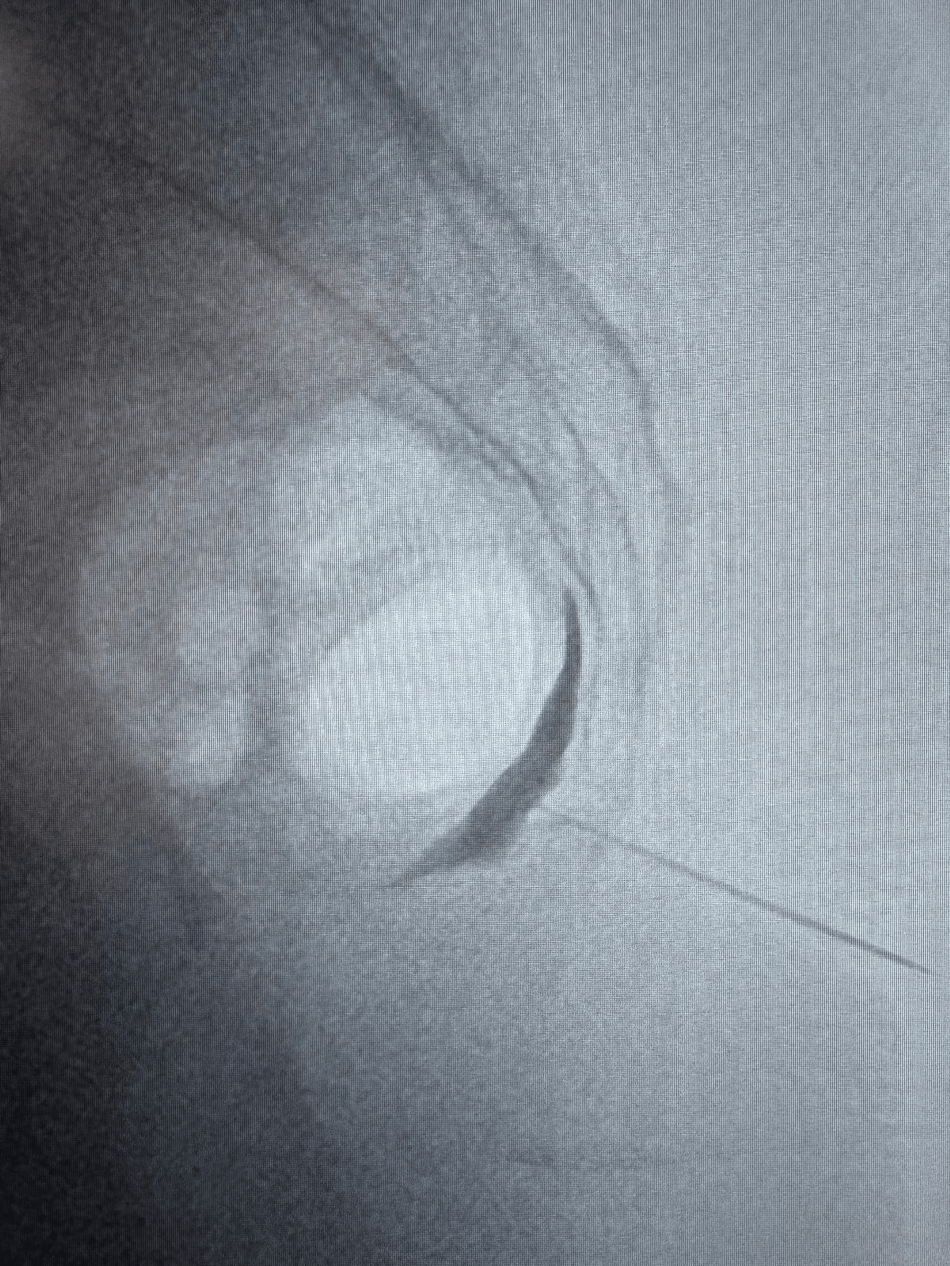
Lateral fluoroscopic view demonstrating needle placement at the anterior aspect of the sacrococcygeal junction (S5-Cx1), with contrast confirming the location of the ganglion impar. The patient provided written informed consent for publication of this image. Image credit: Dr. José Luís Carvalho

A trans-sacrococcygeal approach was used with fluoroscopic guidance. After confirming the position of the sacrococcygeal junction under anteroposterior and lateral fluoroscopic guidance, 1 mL of 2% lidocaine was infiltrated subcutaneously for local anaesthesia. Then, an 88 mm, 22G needle was inserted; its positioning and progression were confirmed with a fluoroscopic lateral view. Once the needle passed the anterior aspect of the sacrococcygeal junction (S5-Cx1), 1 mL of contrast was injected to confirm the correct positioning in the ganglion impar location and to ensure the absence of intravascular spread (Figure 1). Then, we performed a ganglion impar block with local anaesthetic and corticosteroid (3 mL of 2% ropivacaine and 1 mL of 40 mg/mL methylprednisolone). Adverse events were recorded.

In those patients with a previous positive response to ganglion impar block, PRF was performed. The PRF needle positioning technique was similar to the technique mentioned above. A 10 cm, 22G Boston Scientific needle with a 10 mm active tip and Boston Scientific G4 Radiofrequency Generator was used (Boston Scientific Corporation, Marlborough, MA, USA). Sensitive and motor stimulation was performed to enhance the safety and accuracy of the procedure. PRF parameters were 100 V at 42ºC for six minutes. Adverse events were documented.

## Results

After the analysis of all electronic medical records of fluoroscopy-guided procedures performed at the Intervention and Musculoskeletal Rehabilitation Unit, 24 patients fulfilled the inclusion criteria. One patient was excluded since he had died prior to data collection.

In our case series of 24 included patients, eight (33.3%) presented a history of direct trauma to the coccyx, five (20.8%) had degenerative changes or coccygeal hyper/hypomobility or subluxation, and two (8.3%) reported postpartum coccydynia, although 13 (54.2%) had undergone vaginal delivery. Regarding oncological comorbidities, two (8.3%) had a history of pelvic malignancy: one with human papillomavirus (HPV)-associated cervical carcinoma and another with a benign coccygeal tumour that had been surgically excised. On physical examination, all patients (100%) exhibited tenderness over the coccygeal region on external palpation.

Considering the risk factors for developing coccydynia, females comprised 22 patients (91.7%). While obesity has been widely documented in the literature as a risk factor, our case series exhibited BMI values within the normal reference interval, on average 24.2 kg/m² (standard deviation (SD) = 5.3 kg/m², range = 14-38 kg/m²). Regarding psychiatric comorbidities, three (12.5%) had depression, and four (16.7%) had anxiety. 

Additionally, to analyse the effectiveness of an interventional procedure (ganglion impar block and/or PRF) in patients with anxiety and depression, we specifically analysed patients presenting both comorbidities. Anxiety and depression were observed in three of the 24 patients (12.5%), all of whom were female, with a mean age of 32 years (SD = 5.3 years, range = 26-36 years). The mean weight was 63 kg (SD = 14 kg, range = 47-73 kg), and the mean height was 166 cm (SD = 7.2 cm, range = 160-174 cm). The mean BMI was normal, 22.7 kg/m² (SD = 3.8 kg/m², range = 18.4-25.7 kg/m²). Two (66.7%) patients referred a previous history of trauma in that region, and one of them had coccygeal subluxation (33.3%). Two (66.7%) had vaginal deliveries, but only one (33.3%) reported postpartum coccydynia. The mean preprocedural NRS was 8.3 (SD = 1.5; range = 7-10). All patients reported significant pain relief, regardless of the procedure they underwent. The mean NRS at one-month follow-up was 2 (SD = 1.7; range = 0-3), representing an average benefit of 76% pain relief. At three-month follow-up, the mean NRS remained 2 (SD = 1.7; range = 0-3), maintaining a 76% pain relief. Six months after the procedure, the mean NRS was 4.3 (SD = 5.1; range = 0-10), representing a 48% pain reduction. No complications were reported.

Of the 24 included patients in our series, 14 (58.3%) were naïve to any prior interventional procedure and underwent a ganglion impar block. Of these, 12 (85.7%) were female, and two (14.3%) were male. The mean age of these patients was 46.1 years (SD = 18.7 years, range = 26-87 years). The mean weight was 66.4 kg (SD = 23.4 kg, range = 32-130 kg), and the mean height was 162.1 cm (SD = 9 cm, range = 150-185 cm). The mean BMI was 24.9 kg/m², which is at the upper limit of the normal BMI range (SD = 6.8 kg/m², range = 14-38 kg/m²). Two (14.3%) had depression, and three (21.4%) had anxiety. Six (42.9%) reported a previous history of trauma in that region; however, the majority (57.1%) had no trauma history. Nine (64.3%) had clinical records revealing joint dysfunction (e.g., degenerative joint disease; hyper/hypomobility of the sacrococcygeal joint). Of the 12 female patients, seven (58.3%) had vaginal delivery prior to coccydynia, but no causal connection was inferred by the patients; five (41.7%) had no vaginal delivery. The mean baseline NRS was 8.4 (SD = 1.4; range = 6-10). Most patients reported significant pain relief. The mean NRS at one-month follow-up was 3 (SD = 3.4; range = 0-10), representing 64% pain relief. At three-month follow-up, the mean NRS was 3.4 (SD = 3.4; range = 0-10), representing 60% pain relief. Six months after the procedure, the mean NRS was 4.2 (SD = 3.8; range = 0-10), representing a 50% pain reduction. No complications occurred. Patient demographics, clinical characteristics, and NRS scores at baseline and at one, three, and six months following ganglion impar block are summarized in Table 1.

**Table 1:** Demographic Characteristics, Clinical Features, and NRS Scores of Patients Submitted to Ganglion Impar Block. M: Male; F: Female; 0: No; 1: Yes; -: Not Applicable; NRS: Numerical Rating Scale

Gender	Age	Weight (kg)	Height (cm)	BMI (kg/m²)	Depression	Anxiety	Tenderness upon external palpation in the coccygeal region	Trauma in the coccygeal region	Degenerative changes, coccygeal hyper/hypomobility, subluxation	Vaginal delivery	NRS before procedure	NRS at 1 month	NRS at 3 months	NRS at 6 months
F	26	47	160	18.4	1	1	1	0	0	0	7	3	3	3
M	40	130	185	38	0	1	1	1	1	-	9	0	0	0
F	37	61	167	21.9	0	0	1	1	0	1	6	0	0	0
F	50	49	157	19.9	0	0	1	0	0	0	9	0	6	6
F	36	60	163	22.6	0	0	1	1	1	0	9	5	7	8
F	34	69	164	25.7	1	1	1	1	1	1	10	3	3	10
F	53	78	167	28	0	0	1	0	0	1	10	8	8	-
F	87	60	150	26.7	0	0	1	0	0	1	10	2	2	2
F	39	61	154	25.7	0	0	1	0	0	1	7	0	0	0
F	81	66	165	24.2	0	0	1	0	0	1	8	0	0	0
M	60	77	170	26.6	0	0	1	1	1	-	7	6	5	3
F	26	32	153	13.7	0	0	1	0	0	0	10	10	10	10
F	33	50	160	19.5	0	0	1	1	1	0	8	5	4	4
F	43	90	154	37.9	0	0	1	0	0	1	7	0	0	8

Of these 14 naïve patients, two (14.3%) repeated the ganglion impar block, on average six months later, due to pain recurrence. They were all female, with an average age of 34.5 years (SD = 2.1 years). The mean weight was 55 kg (SD = 7.1 kg), and the mean height was 161.5 cm (SD = 2.1 cm). The mean BMI was normal: 21.1 kg/m² (SD = 2.2 kg/m²). They had no previous history of mental health conditions or vaginal delivery. All (100%) had a previous history of trauma and records of sacrococcygeal osteoarthritis. The mean baseline NRS prior to the second ganglion impar block was 7 (range = 5-9). Patients reported significant pain relief; the mean NRS at one-month follow-up was 4 (range = 2-6), representing an average benefit of 43% pain relief. At three-month follow-up, the mean NRS was 5 (range = 2-8), representing a 29% reduction in pain. Six months after the procedure, the mean NRS was 5.5 (range = 2-9), representing a 21% decline in pain intensity.

From the total of 24 patients included in our series, 10 (41.7%) underwent PRF after a positive ganglion impar block. All of these patients were female. The mean age was 44.7 years (SD = 9.8 years, range = 31-63 years). The mean weight was 62.8 kg (SD = 9 kg, range = 49-75 kg), and the mean height was 164.6 cm (SD = 7.5 cm, range = 154-175 cm). The mean BMI was normal, 23.1 kg/m² (SD = 2.1 kg/m², range = 19.5-25.7 kg/m²). One patient (10%) had depression, and one (10%) had anxiety. Two patients (20%) had a previous history of trauma to the coccygeal region. None had records of degenerative or mechanical alterations in the coccyx. Six patients (60%) had vaginal deliveries; two (33.3%) reported the onset of pain after vaginal deliveries that were described as difficult and performed with instruments (forceps or vacuum-assisted deliveries). The mean baseline NRS was 8.5 (SD = 1.4, range = 6-10). Most patients reported significant pain relief; the mean NRS at one-month follow-up was 2.3 (SD = 3.2, range = 0-10), representing an average benefit of 73% pain relief. At three-month follow-up, the mean NRS was 3.2 (SD = 3.4, range = 0-10), representing a 62% reduction in pain. Six months after the procedure, the mean NRS was 4.3 (SD = 3.2, range = 0-10), representing a 49% reduction in pain. No complications occurred. Table 2 summarizes patient-specific demographics, clinical findings, and NRS scores at baseline and at one, three, and six months after PRF. 

**Table 2:** Demographic Characteristics, Clinical Features, and NRS Scores of Patients Submitted to Ganglion Impar PRF. M: Male; F: Female; 0: No; 1: Yes; -: Not Applicable; NRS: Numerical Rating Scale; PRF: Pulsed Radiofrequency

Gender	Age	Weight (kg)	Height (cm)	BMI (kg/m²)	Depression	Anxiety	Tenderness upon external palpation in the coccygeal region	Trauma in the coccygeal region	Degenerative changes, coccygeal hyper/hypomobility, subluxation	Vaginal delivery	NRS before procedure	NRS at 1 month	NRS at 3 months	NRS at 6 months
F	46	61	161	23.5	0	0	1	1	0	0	6	0	0	3
F	37	61	167	21.9	0	0	1	0	0	1	9	5	8	8
F	50	49	157	19.9	0	0	1	0	0	0	9	0	4	5
F	36	73	174	24.1	1	1	1	1	0	1	8	0	0	0
F	63	50	160	19.5	0	0	1	0	0	1	10	10	10	10
F	53	60	158	24	0	0	1	0	0	0	8	3	2	1
F	52	75	175	24.5	0	0	1	0	0	1	10	0	3	5
F	39	61	154	25.7	0	0	1	0	0	1	7	3	3	6
F	31	73	170	25.3	0	0	1	0	0	0	10	1	1	4
F	40	65	170	22.5	0	0	1	0	0	1	8	1	1	1

Of these 10 patients submitted to PRF, two (20%) underwent a second PRF of the ganglion impar, on average 10 months after the first PRF. They were, on average, 43 years old (SD = 4.2 years). The mean weight was 63 kg (SD = 2.8 kg), and the mean height was 165.5 cm (SD = 6.4 cm). The mean BMI was normal: 23 kg/m² (SD = 0.7 kg/m²). They had no previous history of mental health conditions or mechanical or degenerative alterations in the sacrococcygeal joint. One patient (50%) had a vaginal delivery; however, she did not correlate it with the onset of pain. One patient (50%) had a previous history of direct trauma to the sacrococcygeal region. The mean baseline NRS prior to the second procedure was 6.5 (range = 5-8). Patients reported pain relief, with a mean NRS of 3.5 (range = 0-7) at one-month follow-up; the average benefit was a 46% reduction in pain. At three-month follow-up, the mean NRS was 4 (range = 0-8), representing a 38% reduction in pain, which remained the same six months after the procedure.

## Discussion

We included a heterogeneous patient population - covering trauma-related, degenerative, coccygeal hyper- or hypomobility, subluxation, obstetrical, oncological, and psychiatric comorbidities - offering insight into the diverse clinical scenarios of coccydynia. Our findings are consistent with previous reports, reinforcing that coccydynia is a multifactorial condition with both traumatic and non-traumatic aetiologies [1-4]. Similar to what has been described in the literature, in our series, the most frequent cause of coccydynia was direct trauma to the coccyx, reported by one-third (33.3%) of the patients [1]. While obesity has been widely documented in the literature as a risk factor, it is noteworthy that, in our case series, patients’ BMI was normal, on average 24.2 kg/m² [1].

A bidirectional association between chronic coccydynia and anxiety or depression has been pointed out in the literature [5]. In our series, one in six patients presented one of these psychiatric comorbidities. Regardless of the procedure performed, these patients experienced meaningful pain relief (76% reduction in NRS scores during the first three months after the procedure, and 49% at six months), showing a significant response to interventional treatments for chronic coccygeal pain. Contrary to what has been reported in the literature, our findings suggest that patients with anxiety or depression may still benefit from minimally invasive procedures [6]. It seems that psychiatric comorbidities should not be considered a barrier to minimally invasive procedures. However, with only three patients having both conditions, no definitive conclusions can be drawn, and this claim requires confirmation in larger cohorts.

This case series demonstrates methodological rigor in several key areas. All procedures were performed by a single clinician, ensuring procedural consistency. We provided a detailed description of the fluoroscopy-guided ganglion impar block and PRF technique - including needle specifications, anaesthetic mixture, contrast confirmation, and PRF parameters - which facilitates clinical application and reproducibility by other clinicians. Additionally, our findings corroborate prior studies reporting the efficacy of the ganglion impar corticoanesthetic block in coccydynia caused by non-cancer-related pain, and reinforce that, despite the heterogeneous aetiology of the condition, interventional management targeting the ganglion impar can be effective across different underlying causes [7-12]. Furthermore, with the exception of one patient who died prior to the telephone interview, all participants provided complete data for all analysed variables, supporting internal validity. The absence of complications across all procedures further underscores the safety of these interventions.

Although we assessed pain at one, three, and six months to explore the short- and medium-term analgesic effects of fluoroscopy-guided ganglion impar block and PRF, the absence of a control group prevents us from establishing effectiveness and limits us to describing outcomes. In our case series, the ganglion impar corticoanaesthetic block provided meaningful short-term benefit, with an average pain reduction of 64% at one month. As expected, this effect in pain intensity progressively declined to 50% at six months, which is consistent with the transient pharmacological effect of local anaesthetics and corticosteroids. In contrast, PRF yielded a stronger initial response, with a mean pain reduction of 73% at one month, maintaining a clinically significant effect of nearly 50% at six months. PRF may serve as a useful treatment option for patients with coccydynia who have a positive prognostic block. It could be expected that PRF would provide a more durable and stronger analgesic benefit compared to a single block, bearing in mind its neuromodulatory mechanism of action compared with the duration of the anaesthetics and corticosteroids. However, in our case series, six months after the procedure, the effect on pain reduction was practically the same for both procedures. These findings are in line with previous studies reporting the effectiveness of both ganglion impar-targeted interventions for chronic coccydynia, highlighting their role as valuable first-line options when conservative measures fail. Given that chronic coccydynia is uncommon and understudied, even our modest datasets may provide meaningful clinical insight. However, we must note that our series suggests similar outcomes for ganglion impar block and PRF, but the study design does not allow a true comparison: patients were not randomized, PRF was administered only after a positive block, and the cohorts differed in size and baseline characteristics. 

Patients who required a repetition of the ganglion impar block demonstrated less favourable outcomes. In this subgroup, the analgesic effect was less pronounced and shorter-lasting compared with the initial block, with pain relief achieving a 43% reduction at one month, but merely 21% at six months. Similarly, patients who underwent a second PRF procedure also experienced a diminished response compared to the first intervention, although the magnitude of pain reduction was self-reported as relevant (46% and 38% pain reduction at one and six months, respectively). While previous reports have suggested that repeated procedures can provide sustained pain relief, our case series highlights that, when repeated, these interventions may not provide sustained benefit in refractory cases [15]. In patients whose pain recurs after initial improvement, escalation to alternative ablative strategies - such as thermal radiofrequency targeting the ganglion impar or sacrococcygeal nerves, or chemical neurolysis - should be considered to achieve further pain control [16-18]. 

Several limitations should be acknowledged. The retrospective design, small sample size, and reliance on descriptive statistics limit the statistical power and generalizability of the findings. Although patients were described as “refractory to conservative treatments,” details regarding prior therapies - including type, duration, physical therapy protocols, medication regimens, and criteria for treatment failure - were not provided. This heterogeneity in prior management reduces comparability and reproducibility. Functional outcomes were not assessed, and pain scores alone may not fully capture treatment benefit. Future prospective studies are warranted to establish standardized management protocols, ideally stratified according to the underlying aetiology of coccydynia.

## Conclusions

Effective management of chronic coccydynia requires a biopsychosocial approach within a multidisciplinary framework to fully address the complex factors contributing to persistent pain in the coccygeal region. When conservative treatments are insufficient, ganglion impar corticoanaesthetic block and PRF of the ganglion impar may represent valuable initial interventional options. Although both interventions appear to provide meaningful pain relief for at least six months, repeated procedures tend to yield diminishing responses, highlighting the need to consider alternative ablative strategies in cases of recurrence.

Our case series underscores the clinical utility of ganglion impar corticoanaesthetic block and PRF as first-line interventions for chronic coccydynia refractory to conservative treatment. It also emphasizes the importance of further research to establish standardized management protocols and optimal escalation pathways for refractory coccydynia.
